# Icariin reduces cognitive dysfunction induced by surgical trauma in aged rats by inhibiting hippocampal neuroinflammation

**DOI:** 10.3389/fnbeh.2023.1162009

**Published:** 2023-06-07

**Authors:** Lifeng Wang, Guanfa Peng, Li Chen, Mingling Guo, Bingyu Wang, Yunmeng Zhang, Jianshun Zhou, Maolin Zhong, Junming Ye

**Affiliations:** Department of Anesthesiology, The First Affiliated Hospital, Gannan Medical University, Ganzhou, China

**Keywords:** postoperative cognitive dysfunction, Icariin, neuroinflammation, surgery, cognitive impairment, TLR4/NF-κB

## Abstract

Postoperative cognitive dysfunction (POCD) is a common postsurgical complication in elderly individuals, significantly impacting the quality of life of patients; however, there is currently no effective clinical treatment for POCD. Recent studies have shown that Icariin (ICA) has antiaging effects and improves cognitive function, but its effect in POCD has not been studied. In this study, we investigated the influence of ICA on cognitive function and the TLR4/NF-κB signaling pathway in a POCD rat model. We found that ICA reduced surgery-induced memory impairment, decreased hippocampal inflammatory responses, ameliorated neuronal injury in the hippocampus and inhibited microglial activation. In addition, we also observed that ICA inhibited activation of the TLR4/NF-κB signaling pathway. In summary, our research suggest that ICA can ameliorate surgery-induced memory impairment and that the improvements resulting from administration of ICA may be associated with inhibition of hippocampal neuroinflammation. Our research findings also provide insight into potential therapeutic targets and methods for POCD.

## Introduction

Postoperative cognitive dysfunction (POCD) is a prevalent complication in elderly individuals following surgery, manifesting mainly as memory impairment, decreased information processing ability and reduced attention, and is also associated with various negative consequences, such as mood and personality alterations (Lin et al., 2020). According to the International Study of Postoperative Cognitive Dysfunction (ISPOCD), the incidence of POCD in elderly patients (aged over 60) was roughly 25.8% within seven days after surgery and approximately 10% within three months after surgery (Moller et al., 1998). Clinical evidence indicates that POCD may lead to long-term cognitive deficits, seriously decrease the quality of life of patients, prolong hospitalization after surgery, increase medical expenses and impose a substantial burden on both patients and society (Steinmetz et al., 2009). However, the pathogenic mechanism underlying POCD remains poorly understood, and there is no promising therapeutic agent for POCD.

The neuroinflammation caused by surgery plays a vital role in the development of POCD (Safavynia and Goldstein, 2018). Surgical trauma facilitates the proinflammatory state by triggering the release of excessive inflammatory mediators, such as tumor necrosis factor-α (TNF-α), interleukin-1β (IL-1β), and interleukin-6 (IL-6), from the peripheral immune system (Hirsch et al., 2016). These inflammatory cytokines can be transported to the brain through the disrupted blood–brain barrier (BBB), ultimately leading to neuroinflammation (Liu and Yin, 2018). Microglia are innate immune cells in the brain with immune regulation and phagocytosis functions. The increased levels of proinflammatory cytokines in the brain can hyperactivate microglia, inducing further release of proinflammatory cytokines, which causes the inflammatory cascade reaction and leads to a vicious cycle of neuroinflammation (Perry, 2004; Teeling and Perry, 2009). In addition, hyperactivated microglia elicit neurotoxic effects, leading to neuronal damage and influencing the function of neurons associated with learning and memory, ultimately causing cognitive impairment (Garden and Moller, 2006).

**Figure 1:**
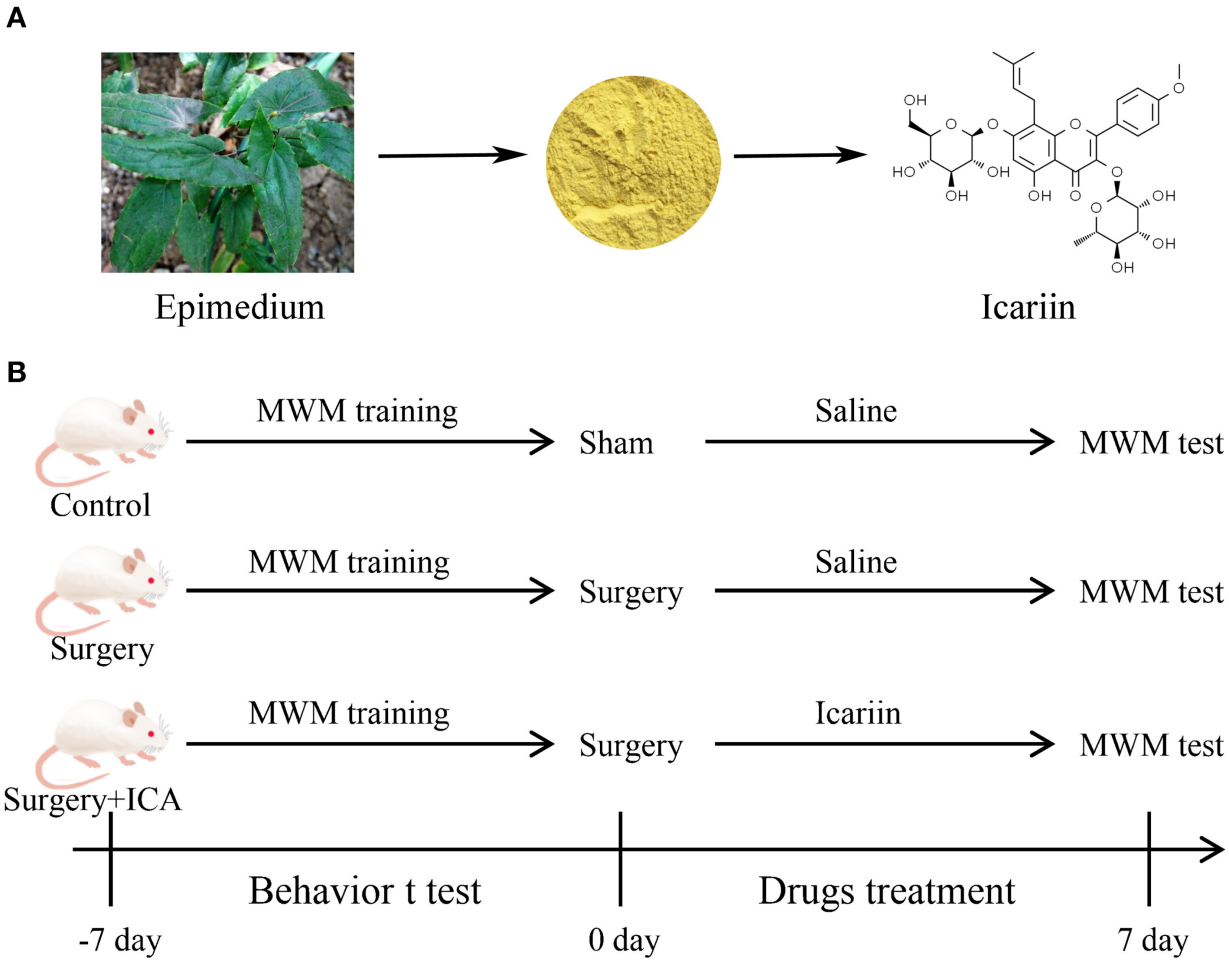
**(A)** Natural sources and chemical structure of Icariin (ICA). **(B)** Diagram of the experimental design. Thirty-six rats were randomly divided into the control, surgery and surgery + ICA groups. All rats were trained on the water maze test for 7 continuous days before the surgery. After modeling, each group was given ICA or saline solution once a day for 7 consecutive days. Subsequently, the water maze test was performed to examine spatial memory.

Icariin (ICA) is a flavonoid compound extracted from the leaves and stems of Herba Epimedii (Figure 1A), a traditional Chinese medicine plant, which has a broad spectrum of therapeutic effects, such as anti-osteoporosis, anti-aging, anti-inflammatory, and antioxidant effects (Wang S. et al., 2021). In the mouse model of diabetic nephropathy induced by streptozotocin (STZ), ICA inhibited the TLR4 signaling pathway, reduced the renal inflammatory response, and played a renoprotective role (Qi et al., 2021). In addition, ICA can improve learning and memory in animals during normal aging. Accumulating studies have provided robust evidence that ICA is a potential therapeutic agent for nervous system diseases (Li et al., 2021). In an Alzheimer's disease (AD) mouse model, ICA administration significantly enhanced learning and memory abilities in APP/PS1 transgenic mice during the Y maze task (Jin et al., 2014). Additionally, ICA decreased the amyloid beta (Aβ) load and amyloid plaque accumulation in the hippocampus of APP transgenic mice by lowering the expression of APP and BACE-1 (Zhang et al., 2014). Liu et al. (2019) found that ICA ameliorated neuroinflammation in the hippocampus by inhibiting the HMGB1/RAGE signaling pathway and played a neuroprotective role by activating the TLR4/NF-κB signaling pathway, resulting in reduced depressive behaviors in mice. Our preliminary experimental results showed that ICA can improve cognitive impairment induced by surgery in rats (Supplementary material); however, it remains unknown whether ICA can decrease neuroinflammation induced by surgery and thereby alleviate postoperative cognitive dysfunction. Consequently, this study aimed to assess the influence of ICA on POCD and further explore the role of the TLR4/NF-κB signaling pathway in the neuroprotective effect of ICA.

## Materials and methods

### Animals and grouping

Twenty-month-old male Sprague–Dawley rats, weighing between 600 and 650 g, were utilized in the experiments. These animals were procured from the Laboratory Animal Center of Gannan Medical University (Ganzhou, China). The rats were housed in a room with controlled humidity and temperature, following a 12-hour light/dark cycle, and provided with unrestricted access to food and water. The experiments were approved by the Ethics Committee of Gannan Medical University and adhered to the guidelines for the Care and Use of Laboratory Animals by the National Institutes of Health (NIH Publications No. 80-23). All experiments were performed such that they minimized both the number of animals used and the suffering of those animals.

To investigate the effect of ICA on the cognitive function of POCD rats, a total of 36 rats were randomly assigned to one of three groups: the control group, surgery group and surgery+ICA group (Figure 1B). The rats in the surgery+ICA group were gavaged with ICA (60 mg/kg, ICA was suspended in saline containing 1% DMSO) (Solarbio, Beijing, China) once a day for 7 consecutive days after surgery, while the rats in the other groups were gavaged with an equivalent volume of normal saline. The dosage of ICA was determined based on previous literature (Jiang et al., 2019).

### Intramedullary fixation of tibia fracture

The rat model of POCD was established by intramedullary fixation of a tibial fracture, following the methods previously reported (Netto et al., 2018). Rats were anesthetized with 2.1% isoflurane, and the knee joint of the left hind limb was then depilated and locally sterilized. A longitudinal incision was made from the knee to the middle third of the tibia, the subcutaneous tissue was dissected and the muscle was removed from the patellar tendon and the tibial periosteum. A hole was drilled in the tibial tuberosity, and then a sterile Kirschner wire (diameter, 1 mm) was inserted into the intramedullary canal to the distal third of the tibia. Subsequently, osteotomy was performed at the junction of the middle and distal third of the tibia, and the incision was then sutured with 4/0 nylon after washing with sterile physiological saline. After surgery, rats were placed on a thermally insulating electric blanket until they regained consciousness. Penicillin was used to prevent infection, and lidocaine gel was applied for analgesia. The rats in the control group had their skin cut under isoflurane anesthesia and then sewn back on without causing a tibial fracture, and received the same postoperative anti-infective and analgesic treatment as the rats in the other groups.

### Morris water maze test

The hippocampal-dependent memory and cognitive abilities were assessed using the Morris water maze, as detailed in a previous study (Feng et al., 2017). In brief, rats were placed in a circular swimming pool filled with warm water in which an escape platform was located 2 cm below the water surface in one quadrant's center. In the acquisition phase, rats were randomly released individually into the water facing the pool wall in each of the four quadrants four times per day for 7 continuous days before surgery. The animals were allowed 90 s per trial to find the platform (10 cm diameter) that was placed below the water surface and were allowed to rest on it for 15 s. If a rat failed to find the platform within the allotted time (90 sec), an experimenter guided the animal to the platform. The time taken to climb onto the platform from entering the water was documented as the escape latency (maximum 90 sec). One week post POCD surgery, rats were subjected to a spatial probe test during which the platform was retracted from the pool. The number of platform crossings, the total time spent in the target quadrant, the time to first platform crossing and the swimming speed were automatically analyzed from video recordings using EthoVision^®^ XT software (Noldus, Wageningen, Netherlands).

### Hematoxylin and eosin (HE) staining and Nissl staining

After the Morris water maze test, the brains of the rats in each group were removed, and the brain tissue containing the hippocampus was then fixed with 4% paraformaldehyde at 4°C overnight. Then, the tissue samples were gradually dehydrated in an alcohol gradient and submerged in xylene. We embedded the samples in paraffin and then sectioned them at a thickness of 5 μM. After deparaffinization and rehydration at room temperature, the slices were stained with HE (Solarbio, Beijing, China) and Nissl staining solution (Solarbio, Beijing, China) in accordance with the manufacturer's instructions. The number of Nissl-positive cells in the hippocampal region was determined by a pathologist blinded to the grouping using a light microscope (Olympus, Tokyo, Japan) at 200 × magnification. For each sample, three sections were taken, and three random fields were observed in each section. The total cell count was then divided by the number of observed fields to quantify the number of Nissl-positive cells in each sample.

### Enzyme-linked immunosorbent assay (ELISA)

Blood samples and hippocampal samples were obtained after the Morris water maze test. The concentrations of TNF-α, IL-1β, and IL-6 in the serum and hippocampus were determined using enzyme-linked immunosorbent assay (ELISA) kits (Elabscience, Wuhan, China) according to the manufacturer's protocol. The optical density (OD) value of each well was measured by an ELISA plate reader (Thermo Scientific, Waltham, MA, USA) at 450 nm. The experiments were repeated three times to allow calculation of mean values.

### Western blot

Hippocampal tissues were homogenized and extracted by utilizing RIPA lysis buffer (Applygen Technology, Beijing, China), which contained protease inhibitor (TransGen Biotechnology, Beijing, China) and phosphatase inhibitor (TransGen Biotechnology, Beijing, China). The total protein concentration in the supernatant was measured with a BCA protein assay kit (Solarbio, Beijing, China). Equal amounts of protein (50 μg/lane) were separated by sodium dodecyl sulfate-polyacrylamide gel electrophoresis (SDS–PAGE), and subsequently transferred to polyvinylidene difluoride (PVDF) membranes (Millipore, Bedford, MA, USA). Next, the membranes were blocked with 5% BSA solution at room temperature for 2 hours and then incubated with primary antibodies specific for the following proteins overnight at 4°C: TLR4 (1:750, Proteintech Cat# 19811-1-AP), p-NF-κB p65 (1:1,250, Affinity Biosciences Cat# AF2006), NF-κB p65 (1:2,500, Proteintech Cat# 10745-1-AP) and GAPDH (1:20,000, Proteintech Cat# 10494-1-AP). After five washes with TBST, the membranes were incubated with the corresponding HRP-conjugated secondary antibodies (1:2,000, Proteintech Cat# SA00001-2) at room temperature for 1 hour. Finally, the membranes were visualized using ECL kits (Epizyme Biotechnology, Shanghai, China), and images were acquired by a chemiluminescent gel imaging system (ChemiDoc XRS+, Bio-Rad, PA, USA). Densitometric analysis of the protein bands was performed with Image Lab software.

### Immunohistochemistry (IHC)

Rat brain samples were preserved using 4% paraformaldehyde, encased in paraffin, and sectioned into 5 μm-thick slices. After paraffin dewaxing and hydration, the slices were incubated with 3% H_2_O_2_ at room temperature for 10-15 min to inhibit endogenous peroxidase activity in the tissues. Then, the slices were subjected to antigen repair with 0.01 M sodium citrate using the thermal repair method in a microwave. Goat serum (10%) was used to block the nonspecific binding sites at 37°C for 30 min. The slices were incubated with the primary antibody against IBA-1 (1:500, Proteintech Cat# 10904-1-AP) at 4°C overnight and then incubated with goat anti-rabbit HRP-linked secondary antibody (1:1,000, Proteintech Cat# SA00001-2) at 37°C for 60 min. Then, the slices were coated with DAB buffer (Zhong Shan Golden Bridge Biotechnology, Beijing, China), and hematoxylin was used to counterstain nuclei. Finally, the hippocampal region was observed under a bright field fluorescence microscope (Carl Zeiss, Oberkochen, Germany) at 200 × magnification. An experimenter who was unaware of the sample identities counted the IBA-1-positive cells using ImageJ software. The number of IBA-1-positive cells from three sections (three visual fields per section) were averaged for each animal and converted to cells/field.

### Statistical methods

All the data are shown as the mean ± standard deviation (SD). GraphPad Prism 8.0 software was used for statistical analysis. One-way analysis of variance (ANOVA) followed by the Tukey–Kramer multiple comparisons test were used to evaluate the significance of differences. *P* < 0.05 indicated a statistically significant difference.

## Results

### ICA improves cognitive impairment of POCD rats

**Figure 2:**
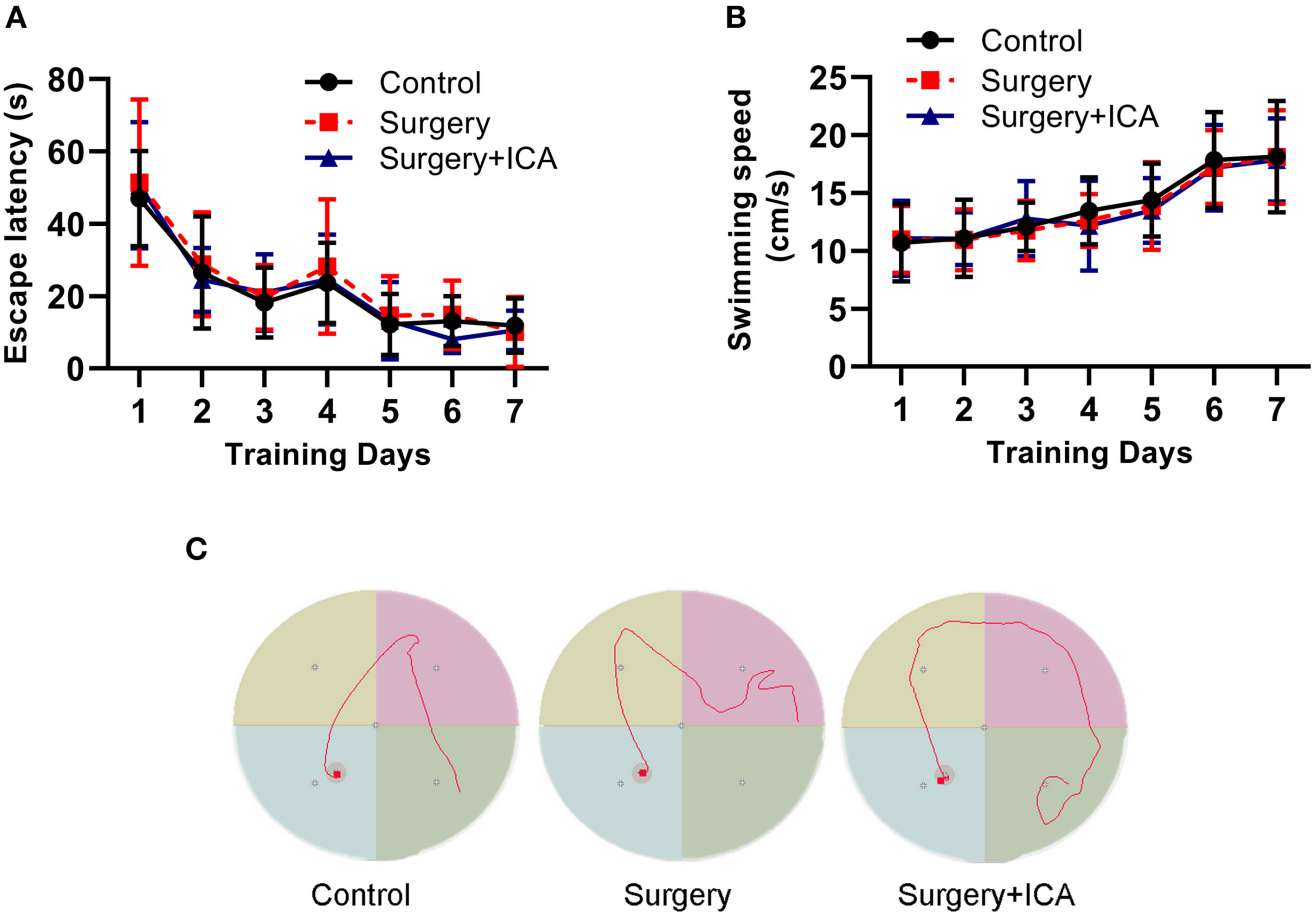
Performance of rats in the training phase of the water maze test. **(A, B)** Rats were trained to find the platform for 7 continuous days preoperatively, and there was no significant difference in the escape latency and swimming speed among the groups. **(C)** Results of motion trials of rats after 7 days of training in the Morris water maze test. *n* = 12 per group.

**Figure 3:**
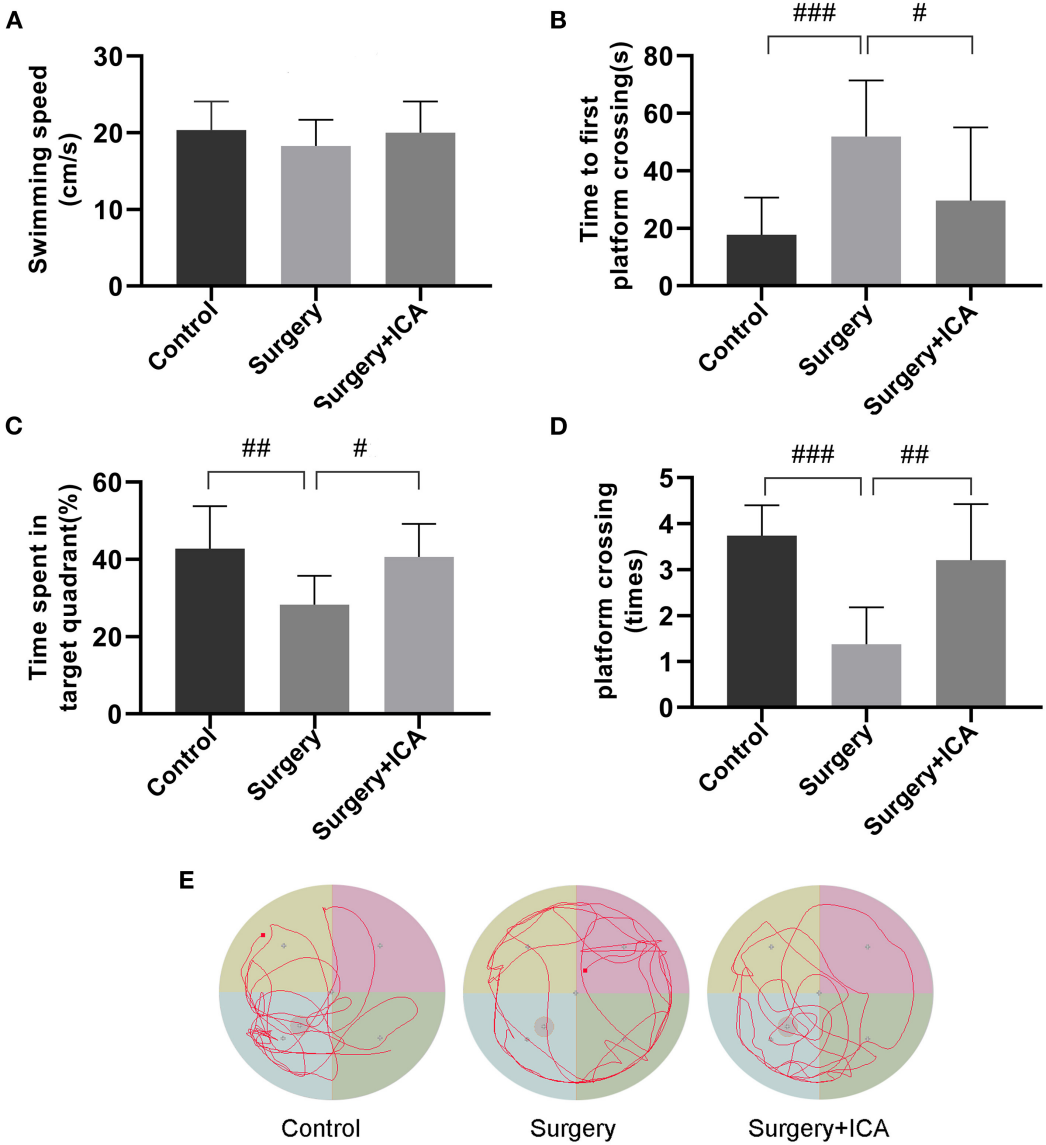
ICA improves cognitive impairment in POCD rats. **(A–D)** In the spatial probe test, swimming speed, time to first platform crossing, the time spent in the target quadrant and the number of platform crossings were used to assess motor function and memory ability by analyzing the motion trajectory. **(E)** The motion trajectory of rats in each group. The data are expressed as the means ± standard deviations; ^#^*p* < 0.05, ^*##*^*p* < 0.01, ^*###*^*p* < 0.001. *n* = 12 per group.

To test whether ICA improves cognitive function in POCD rats, the Morris water maze test was conducted. In the acquisition phase, all rats could find the platform after training and showed improvement in spatial learning and memory over time. There was no significant difference in the escape latency or swimming speed in the acquisition phase among the groups (Figures 2A–C), indicating that the rats in each group had the same baseline learning and memory ability. The spatial probe test (Figure 3E) was conducted on the 7th day after surgery, and the swimming speed of the rats did not differ significantly among the groups (Figure 3A), suggesting that fracture surgery did not affect the motor function of the rats. In comparison to the control group, both the time spent in the target quadrant and the number of platform crossings of rats in the surgery group were notably reduced (Figures 3C, D), and the time to first platform crossing of rats in the surgery group was significantly increased (Figure 3B), indicating that intramedullary fixation of tibia fracture resulted in cognitive dysfunction in rats, thus successfully establishing a POCD rat model. The effect of surgery was reversed by ICA treatment (Figures 3B–D), suggesting that ICA treatment improved memory capabilities in aged POCD rats. Taken together, these findings propose that ICA can alleviate cognitive impairment induced by surgery.

### ICA alleviates neuroinflammation in hippocampus of POCD rats

**Figure 4:**
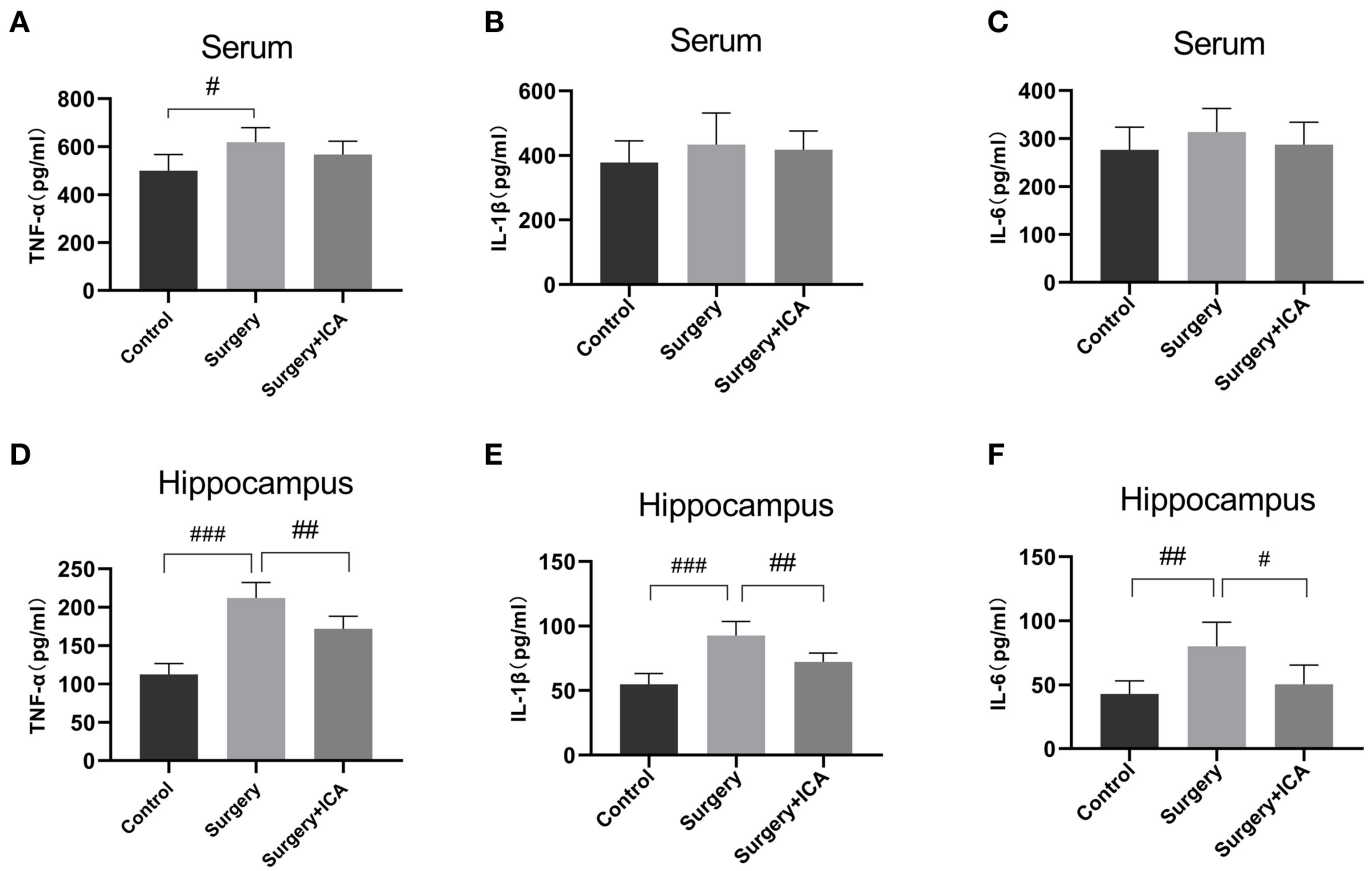
ICA alleviates neuroinflammation in the hippocampus of POCD rats. **(A–C)** The levels of serum inflammatory cytokines TNF-α, IL-1β and IL-6 among the groups were analyzed using an ELISA. **(D–F)** The levels of neuroinflammation were assessed by detecting the expression of TNF-α, IL-1β and IL-6 in the hippocampus. Data are expressed as the means ± standard deviation; ^#^*p* < 0.05, ^*##*^*p* < 0.01, ^*###*^*p* < 0.001. *n* = 6 per group.

Neuroinflammation associated with surgical trauma is the key pathogenic factor in POCD. Surgical stress leads to an increase in the levels of peripheral inflammatory factors, which subsequently leads to an increase in the level of inflammation in the hippocampus. Thus, we assessed the levels of inflammatory factors in the peripheral blood and hippocampus one week postsurgery. The serum concentrations of TNF-α rose in the surgery group (Figure 4A), while the serum levels of IL-1β and IL-6 were not significantly different among the groups (Figures 4B, C), indicating that peripheral inflammation returned to normal levels 7 days after surgery, and ICA had no significant impact on the normal peripheral inflammation level (Figures 4A–C). However, on the 7th day after surgery, the level of inflammation in the hippocampus remained high in the surgery group, while ICA treatment considerably reduced the levels of TNF-α, IL-1β, and IL-6 in the hippocampus of rats (Figures 4D–F). These data indicate that ICA can reduce neuroinflammation in the hippocampus.

### ICA ameliorates neuronal damage in hippocampus of POCD rats

**Figure 5:**
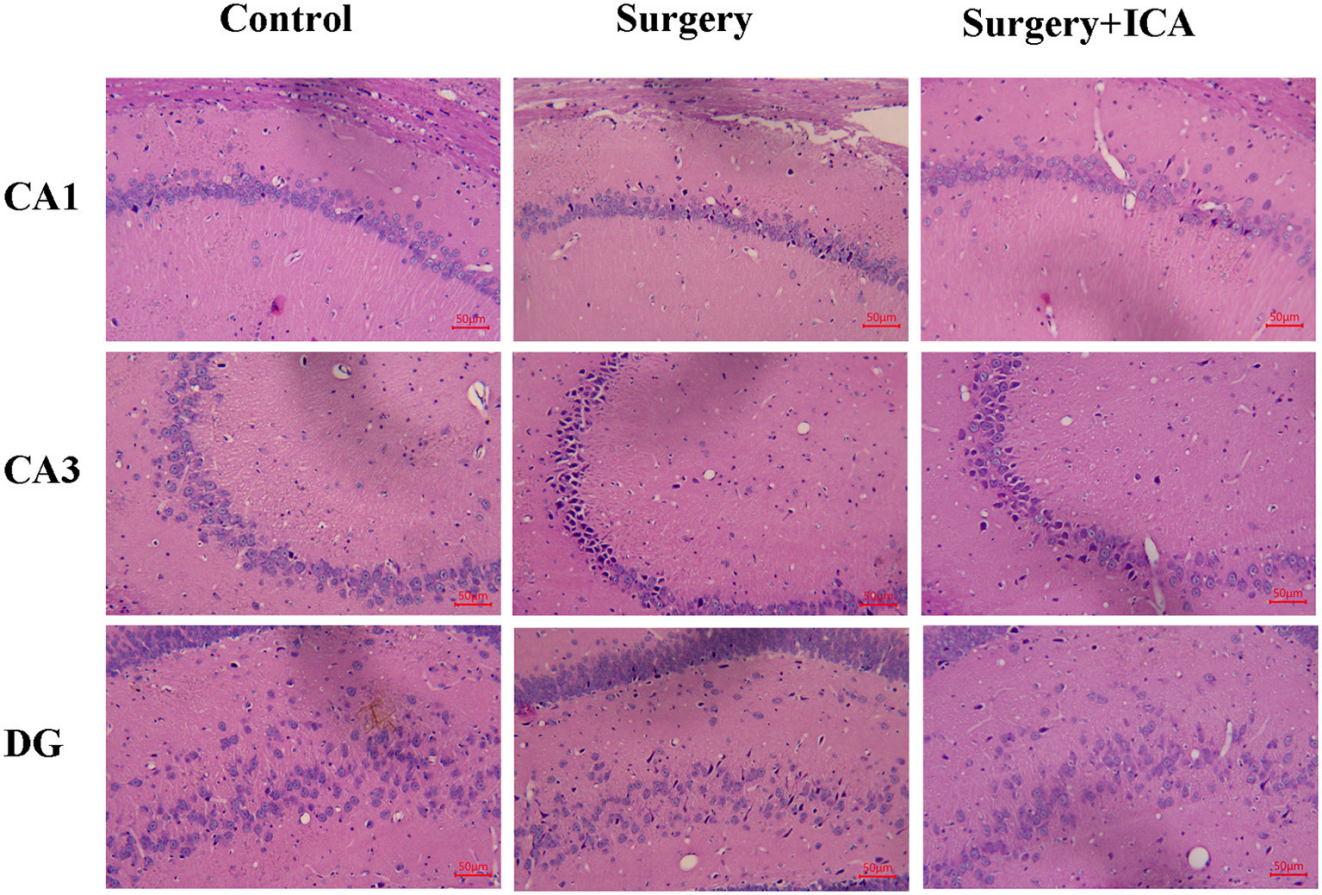
ICA ameliorates neuronal damage in the hippocampus of POCD rats. After HE staining, the morphological changes in the CA1, CA3 and DG regions of the rat hippocampus were observed under an optical microscope (200 × ).

**Figure 6:**
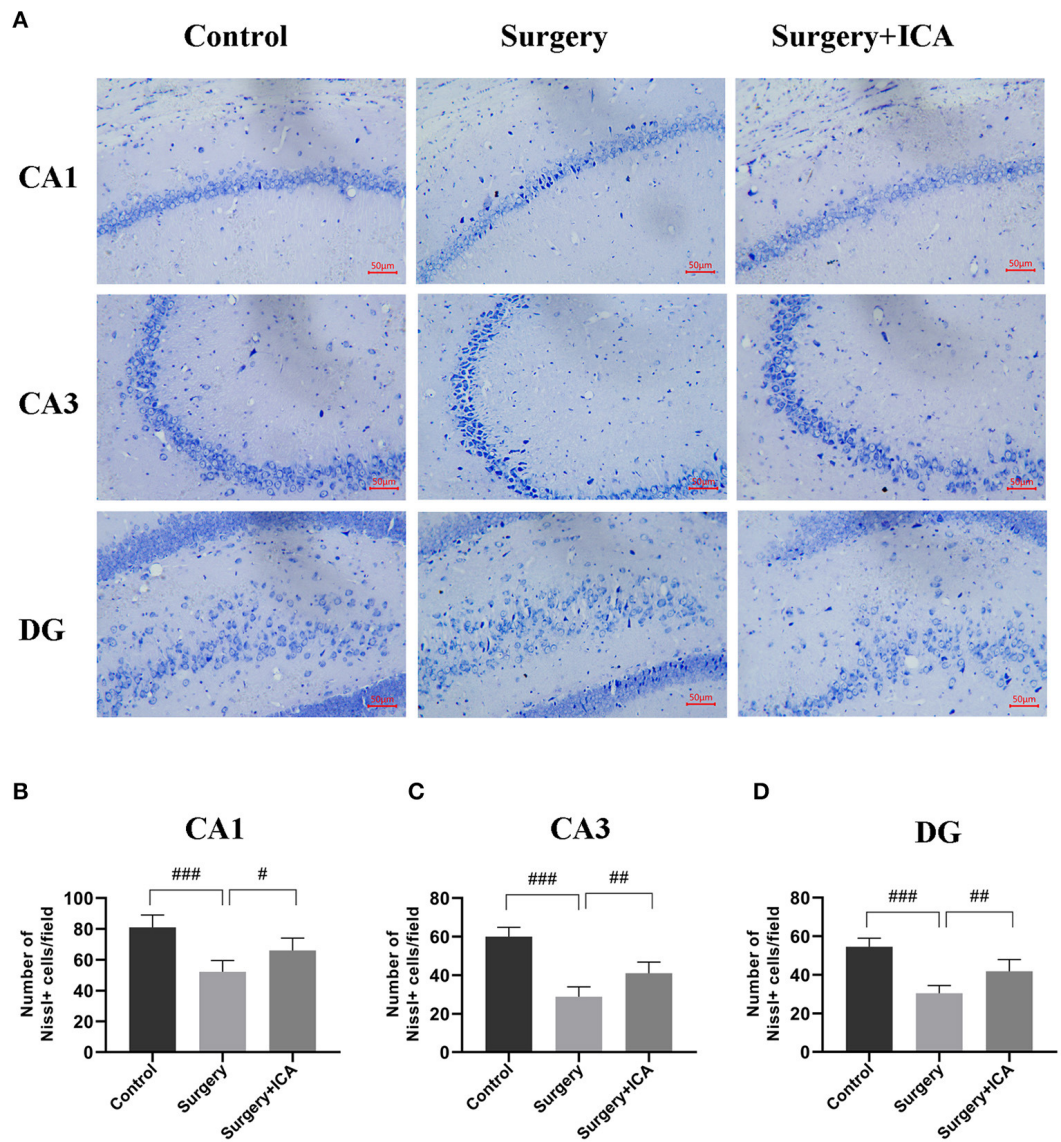
ICA ameliorates neuronal damage in the hippocampus of POCD rats. **(A)** Nissl staining was applied to detect the histopathological changes in hippocampal neurons in CA1, CA3 and DG (scale bar = 50 μm). **(B–D)** The number of Nissl-positive cells in CA1, CA3 and DG were quantified. The data are expressed as the means ± standard deviations; ^#^*p* < 0.05, ^*##*^*p* < 0.01, ^*###*^*p* < 0.001. *n* = 6 per group.

The HE staining results revealed that the neurons in the CA1, CA3 and DG regions in the control group were arranged in an orderly pattern, evenly stained, and regular in shape, whereas those in the surgery group were scattered, irregular, and unevenly stained (Figure 5). After ICA treatment, the neurons were regularly shaped, and the staining was uniform, indicating that the pathological damage to neurons was reversed by ICA (Figure 5). To evaluate neuronal survival in the CA1, CA3 and DG regions of the hippocampus, Nissl staining of brain sections was performed (Figure 6A). Nissl staining showed that the density of neurons in these hippocampal regions was normal in the control group, while the number of Nissl-positive cells obviously decreased in the surgery group (Figures 6B–D). However, ICA treatment significantly attenuated this effect (Figures 6B–D).

### ICA inhibits the activation of microglia of POCD rats

**Figure 7:**
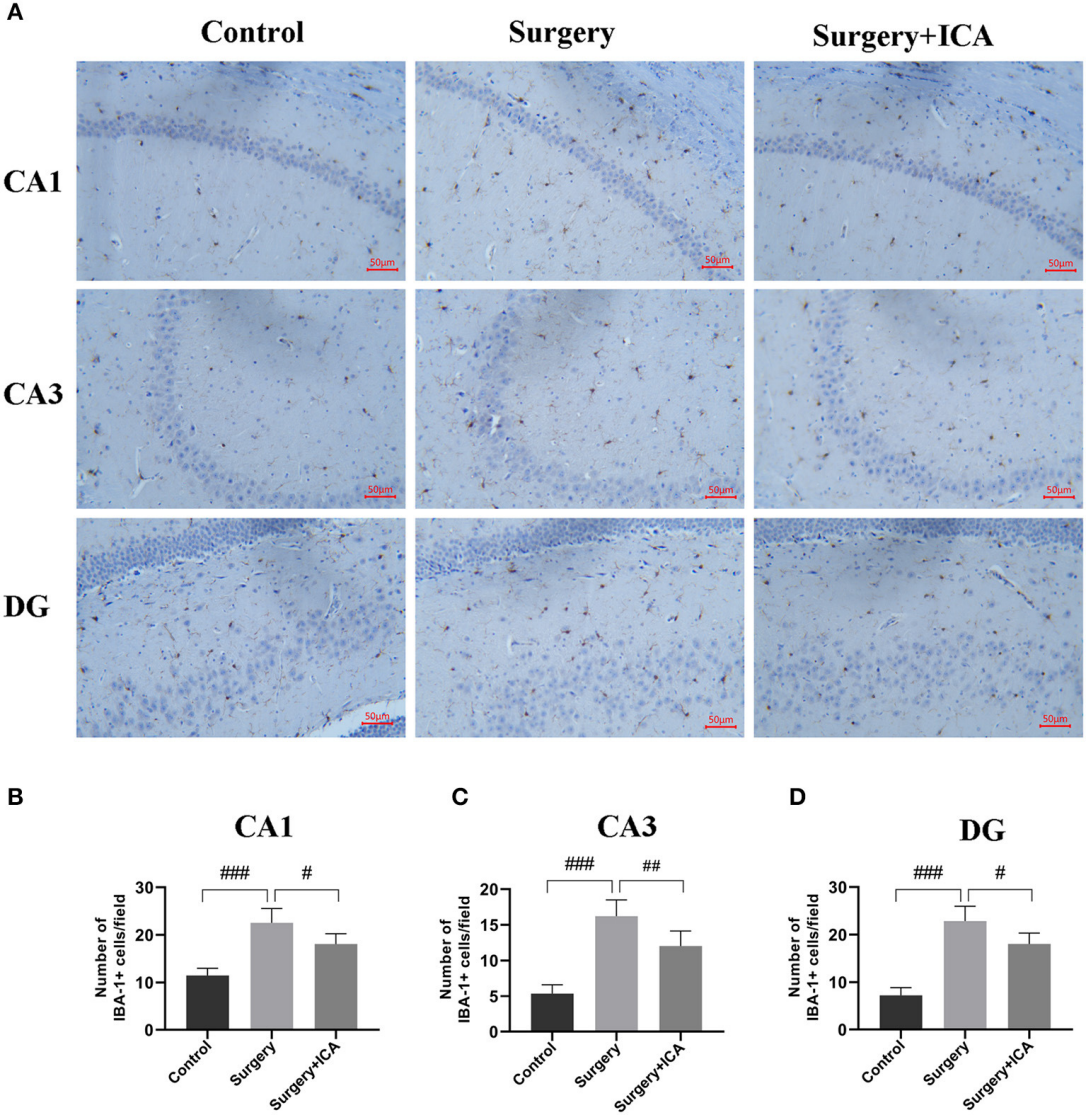
ICA inhibits the activation of microglia in POCD rats. **(A)** The activation of microglia in the CA1, CA3 and DG regions of the hippocampus was evaluated by immunohistochemistry (scale bar = 50 μm) in each group. **(B–D)** The number of IBA-1-positive cells in CA1, CA3 and DG was counted using ImageJ software. The data are expressed as the means ± standard deviations; ^#^*p* < 0.05, ^*##*^*p* < 0.01, ^*###*^*p* < 0.001. *n* = 6 per group.

Microglia have a crucial function in mediating neuroinflammation and neuronal damage in the hippocampus. Therefore, we labeled microglia with IBA-1 7 days postsurgery. As shown in Figure 7A, the number of microglia labeled with IBA-1 in the hippocampus of rats from the surgery group significantly increased. In addition, the number of endpoints and branch length of microglia in the surgery group decreased (Supplementary Figures 1B, C), indicating that microglia were activated in rats in the surgery group. After ICA treatment, the number of IBA-1-tagged microglia decreased (Figures 7B–D), the number of endpoints and branch length of microglia increased (Supplementary Figures 1B, C), indicating that the activation of microglia was inhibited (Figures 7B–D). These data suggest that ICA treatment can inhibit the activation of microglia and thus protect neurons in the hippocampus.

### ICA inhibits the activation of TLR4/NF-κB signaling pathway

**Figure 8:**
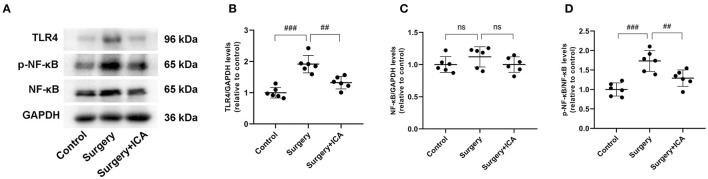
ICA inhibits the activation of the TLR4/NF-κB signaling pathway. **(A)** The protein expression levels of TLR4, NF-κB p65, and p-NF-κB p65 were analyzed using Western blotting. **(B)** The ratio between the optical density value of TLR4 versus GAPDH in the dentate gyrus. **(C)** The ratio between the optical density value of NF-κB versus GAPDH in the dentate gyrus. **(D)** The ratio between the optical density value of p-NF-κB versus the total NF-κB in the dentate gyrus. The data are expressed as the means ± standard deviations; ^*##*^*p* < 0.01, ^*###*^*p* < 0.001. *n* = 6 per group.

The TLR4/NF-κB signaling pathway is closely related to neuroinflammation in central nervous system diseases. We evaluated the expression of proteins associated with the TLR4/NF-κB signaling pathway (Figure 8A). In comparison to the control group, the protein levels of TLR4 and phosphorylated NF-κB in rats in the surgery group increased (Figures 8B–D), suggesting that surgical stimulation activated the TLR4/NF-κB signaling pathway, consistent with previous studies (Shi et al., 2020). In contrast, ICA treatment decreased the protein levels of TLR4 and phosphorylated NF-κB (Figures 8B–D), indicating that the activation of the TLR4/NF-κB signaling pathway was likely inhibited by ICA in POCD rats, which may ultimately inhibit microglia.

## Discussion

In this research, we investigated the effect of ICA on the cognitive function of POCD rats and the potential mechanism underlying this effect. Our research showed that ICA reduced the level of cognitive impairment induced by surgery, which was manifested by the increase in the number of platform crossings, the time spent in the target quadrant and in the decrease in the time to first platform crossing after ICA treatment in the water maze test. ICA also lowered the level of inflammatory factors in the hippocampus. HE staining, Nissl staining and immunohistochemistry showed that ICA ameliorated neuronal damage in the hippocampus and inhibited the activation of microglia. More importantly, we also found that ICA inhibited the activation of the TLR4/NF-κB signaling pathway in the hippocampus.

Currently, surgical trauma is believed to be the main cause of POCD (Saxena and Maze, 2018); thus, surgical trauma was used to establish a POCD model in this study. In addition, it was found that POCD was more common in patients who underwent clinical fracture surgery (Uzoigwe et al., 2020). To better simulate the clinical characteristics, we used intramedullary fixation of tibial fractures to induce POCD in this experiment. The Morris water maze is a classical method for evaluating the cognitive function of rodents. In this study, we conducted a navigation experiment with the Morris water maze test before surgery. Rats were tested four times a day for seven consecutive days to ensure that all rats in each group could find the platform and had the same spatial memory ability in the water maze test. On the 7th day after surgery, a spatial probe test was conducted on rats in each group. The findings revealed that the number of platform crossings and the time spent in the target quadrant were decreased and the time to first platform crossing was increased after surgical trauma stimulation, indicating that surgical stress caused cognitive dysfunction in rats and that the POCD model was successfully established. However, after ICA was given to POCD rats, the number of platform crossings and the time spent in the target quadrant increased, and the time to first platform crossing decreased in the water maze test, indicating that ICA can improve cognitive function in POCD rats.

Previous reports have indicated a close relationship between neuroinflammation and cognitive dysfunction caused by surgery (Subramaniyan and Terrando, 2019; Wang J. et al., 2021). To investigate whether the improvement in cognitive function after administration of ICA in aged POCD rats was related to the inhibition of neuroinflammation, we measured the levels of proinflammatory cytokines such as TNF-α, IL-1β and IL-6 in the peripheral blood and hippocampus. It is believed that surgical stress leads to increases in the levels of peripheral inflammatory factors, which can enter the central nervous system through the damaged BBB, leading to increased neuroinflammation (Saxena et al., 2019). Our study showed that the levels of peripheral inflammatory factors were basically restored to the baseline levels 7 days after surgery, while hippocampal inflammation remained high. ICA treatment did not reduce the baseline level of peripheral inflammation but did reduce the high level of neuroinflammation in the hippocampus, suggesting that ICA inhibited inflammation in the hippocampus. Notably, ICA can also reduce oxidative stress to accelerate fracture healing and promote postoperative recovery (Gurbuz et al., 2019), which may be another reason that ICA ameliorated cognitive impairment induced by surgery.

Accumulating evidence has demonstrated that activation of microglia can aggravate neuroinflammation (Li et al., 2020). Normally, microglia are in an inactive state. However, microglia can be activated during inflammation and BBB destruction. On the one hand, activated microglia can secrete numerous inflammatory factors, such as TNF-α, IL-1β and IL-6, and exacerbate neuroinflammation; on the other hand, activated microglia are neurotoxic and can cause damage to neurons (Chen et al., 2019; Wang Z. et al., 2021). In this study, we examined the morphology and number of neurons in the CA1, CA3 and DG regions of the hippocampus and the activation of microglia. Our results showed that surgery induced the activation of microglia and caused damage to neurons, and ICA reversed this change, consistent with the reduction in hippocampal neuroinflammation.

In addition, we observed that ICA inhibited the TLR4/NF-κB signaling pathway. In the central nervous system, TLR4 is expressed by microglia, astrocytes and neurons (Islam et al., 2022). Activation of TLR4 promotes NF-κB nuclear translocation, which subsequently regulates the mRNA transcription of various proinflammatory mediators, including TNF-α, IL-1β and IL-6, aggravating central nervous inflammation (Zhou et al., 2020). In addition, the TLR4/NF-κB signaling cascade promotes microglial polarization toward the M1 phenotype, which has potent phagocytic properties and is proinflammatory (Ebrahim Soltani et al., 2022). Therefore, the TLR4/NF-κB signaling pathway is crucial in microglia activation and neuroinflammation. Our research showed that intramedullary fixation of tibial fracture activated the TLR4/NF-κB signaling pathway, aligning with prior research findings (Wang et al., 2013; Lu et al., 2015). After treatment with ICA, the TLR4/NF-κB signaling pathway was inhibited. Therefore, it is speculated that ICA may inhibit the release of proinflammatory factors and ameliorate POCD by inhibiting the TLR4/NF-κB signaling pathway. However, a previous study showed that ICA played a neuroprotective role and reduced depressive behavior in mice by activating the TLR4/NF-κB signaling pathway, which contradicts our findings (Liu et al., 2019). These contradictory results may be due to the TLR4/NF-κB pathway playing different roles in different stages of neuroinflammation. In the early stage of neuroinflammation, the TLR4/NF-κB signaling pathway can promote inflammation, while in the late stage of neuroinflammation, the TLR4/NF-κB signaling pathway plays a neuroprotective role and promotes the regeneration of neurons.

This study had several limitations. First, this study solely explored the expression of the TLR4/NF-κB signaling pathway in the rat hippocampus, without assessing its presence in neurons or glial cells. As reports have indicated that the TLR4/NF-κB signaling pathway exists in both neurons and neuroglia, future research should explore the expression of the TLR4/NF-κB signaling pathway in neurons and glial cells of the rat hippocampus by using double immunofluorescence staining. Second, our assessment of inflammation caused by surgery was limited to the hippocampus. However, surgery may have different effects on inflammation in different regions of the brain, such as the striatum, prefrontal cortex, and amygdale (Hovens et al., 2015). Future research should focus on the potential impact of surgery on other brain regions. Third, the present investigation primarily centered on early postoperative cognitive performance, and further research is needed to observe long-term cognitive function. Finally, in this study, we only used male rats, and future research should further investigate the effects of Icariin on postoperative cognitive dysfunction and neuroinflammation in female rats.

## Conclusion

**Figure 9:**
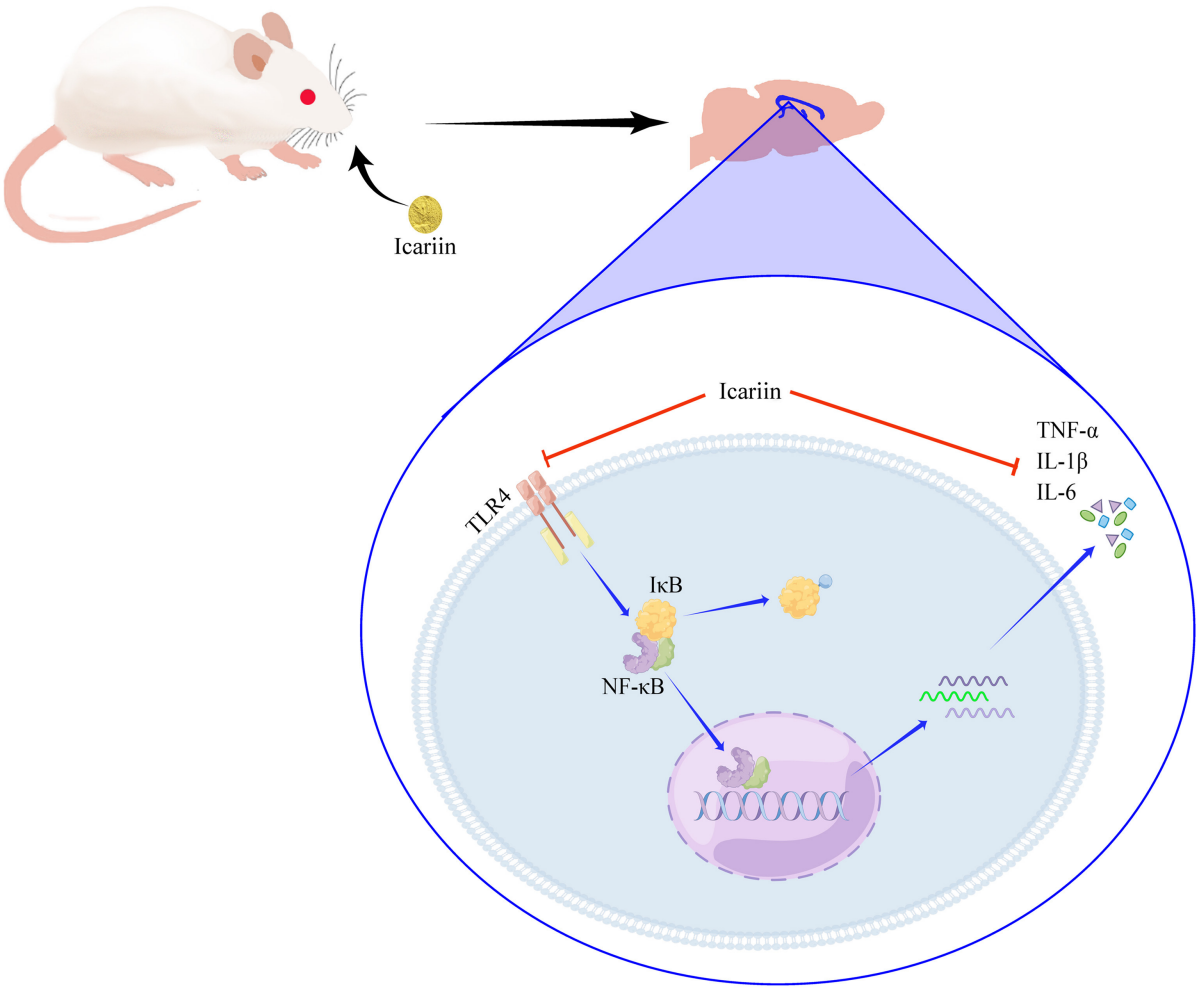
ICA attenuated neuroinflammation by inhibiting the TLR4/NF-κB signaling pathway.

In conclusion, our study showed that ICA can decrease cognitive dysfunction by ameliorating neuroinflammation in aged POCD rats. Moreover, the underlying mechanisms may involve the suppression of the TLR4/NF-κB signaling pathway (Figure 9). ICA may be a promising drug for the treatment of postoperative cognitive dysfunction in elderly patients.

## Data availability statement

The raw data supporting the conclusions of this article will be made available by the authors, without undue reservation.

## Ethics statement

The animal study was reviewed and approved by Ethics Committee of Gannan Medical University.

## Author contributions

JY and MZ contributed to the conception and designed the research study. LW and GP wrote the manuscript, did the HE and Nissl staining, and did the Western blots. JY revised the manuscript. MZ, LC, and BW did the animal caring and surgery. YZ and JZ performed the MWM. BW and YZ did the Elisa assay. JZ and MG did the immunohistochemistry. JY, LW, and GP did the data collection and conducted the statistical analysis. All authors participated in manuscript revision, proofreading, and approved the submitted version criteria.
