# Supplementary material for: Icariin reduces cognitive dysfunction induced by surgical trauma in aged rats by inhibiting hippocampal neuroinflammation
Source: Front Behav Neurosci. 2023 Jun 7;17:1162009. doi: 10.3389/fnbeh.2023.1162009 (PMC10282654; doi:10.3389/fnbeh.2023.1162009)
Supplement: Supplementary file 1 [file Data_Sheet_1.docx]

Supplementary Material

**ICA Improves Cognitive Impairment Induced by Surgery**

**Methods**

**Grouping and Icariin Treatment**

Aged male Sprague-Dawley rats, 20 month old and weighing 600-650 g, were used for the experiments. To investigate the effect of Icariin(ICA) on the cognitive function of POCD rats, 30 rats were randomly divided into control group, surgery group, surgery+ICA(30)( gavaged with ICA 30 mg/kg), surgery+ICA(60)( gavaged with ICA 60 mg/kg), surgery+ICA(120)( gavaged with ICA 120 mg/kg). Rats in drug treatment group were gavaged with ICA (Solarbio, beijing, China) once a day for 7 consecutive days after surgery, while the other groups were gavaged with an equivalent volume of normal saline. The administration of ICA is based on previous studies(Jiang et al., 2019).

**OpenField Test**

Open field test (OFT) was performed as described in previous studies with modifications(Lu et al., 2019). The open field was carried out in a black square box (100 × 100 × 40 cm). The rats were gently placed into the center of the box under dim lighting and were observed for 5 min. The rats in the box was monitored automatically by a video camera, and the behavioral trajectory was analyzed with EthoVision® XT behavior tracking system software (Noldus, Wageningen, Netherlands). The total distance traveled in the box were analyzed to assess the locomotor activity of rats. Between trials the floor of the open field box was wiped with 70% ethanol solution to remove the scent marks.

**Fear Conditioning Test**

The fear conditioning test (FCT) was employed following the protocol described in previous studies(Liu et al., 2021). One day prior to the anesthesia/surgery, rats were trained for fear conditioning to establish long-term memory. Each rat was placed in the conditioning chamber to acclimate for 2 min, and then conditional stimuli (20-s, 70-dB tone auditory cue, 25-s contextual interval) and unconditional stimuli (2-s and 0.70-mA electrical footshock) were repeated for six times. The intervals between two pairs of stimuli were random and ranged from 45 to 60 s. The test phase of the FCT consists of a context test (measure hippocampus-dependent memory) and a tone test (measure hippocampus-independent memory). The rats were allowed in the same conditioning chamber without any stimulus for 5 min during the context test, and 2 h after the context test, the tone test was performed. Rats were placed in a novel chamber which was different from the previous conditioning chamber with the sound stimulus (70 dB, 3 min) but not any footshock stimulus for 5 min. The freezing time during each test was recorded and analyzed by the EthoVision® XT behavior tracking system software (Noldus, Wageningen, Netherlands).

**Statistical Analysis**

All the data are shown as mean ± standard deviation (SD). GraphPad Prism 8.0 software was used for statistical analysis. One-way analysis of variance (ANOVA) was used to evaluate statistical differences followed by Tukey-Kramer multiple comparisions test. Shapiro-Wilk test was used for normality test before statistical analysis. P<0.05 was considered as a statistically significant difference

**Results**

**Surgery or Icariin do not Affect the Locomotor Activity of Rats**

In the OFT, there were no significant differences in total distance traveled by the rats between control group and surgery group at all the preoperative and postoperative time points (**Figure 1A,B,C**). There were a also no significant differences in total distance traveled by the rats between surgery group and surgery+ICA group at all the preoperative and postoperative time points (**Figure 1A,B,C**). These data suggested that the locomotor activity of rats were not affected by sugery or ICA treatment.


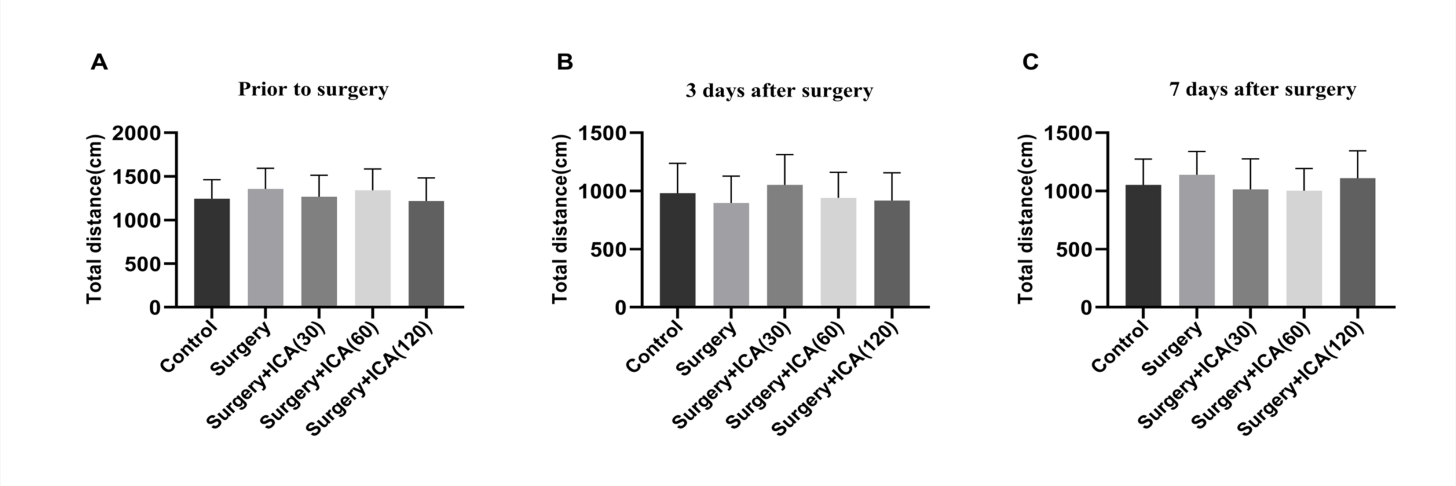


**Supplementary Figure 1**. Surgery or Icariin do not affect tthe locomotor activity of rats. In the OFT, (**A-C**)surgery do not affect the locomotor activity of rats on 1 day prior to surgery and postoperative days 3 and 7, Icariin also do not affect the locomotor activity of rats on 1 day prior to surgery and postoperative days 3 and 7. The data are plotted as the mean ± standard deviation for each group (n = 6 per cohort).

**Surgery Caused Cognitive Impairment and Icariin can Improve Cognitive Impairment of POCD Rats**

In the FCT, the freezing time in the context test and the tone test of the training phase of fear conditioning showed no significant differences between the groups (**Figure** **2A,D**), indicating that the baseline learning and memory abilities of the groups were equal. In the tone test of FCT, there was no significant difference in the freezing time among five groups (**Figure 2E,F**), which suggested that hippocampus-independent memory was not damaged by surgery. Then, in the context test of FCT, compared to the control group, rats in the surgery group decreased the freezing time on postoperative days 3 (**Figure 2B**) and 7(**Figure 2C**). On postoperative days 3, 30mg/kg ICA treatment increased the freezing time but no statistical significance (**Figure 2B**), however, the freezing time were significantly increased with the treatment of 60mg/kg ICA(p=0.04, Fig. 2B) and 120 mg/kg ICA (**Figure 2B**). On postoperative days 7, the freezing time were all significantly increased with the treatment of 30mg/kg ICA, 60 mg/kg ICA and 120mg/kg ICA(**Figure 2C**). These data indicated that ICA treatment can attenuate the impairment of hippocampus-dependent learning and memory in rats with POCD.


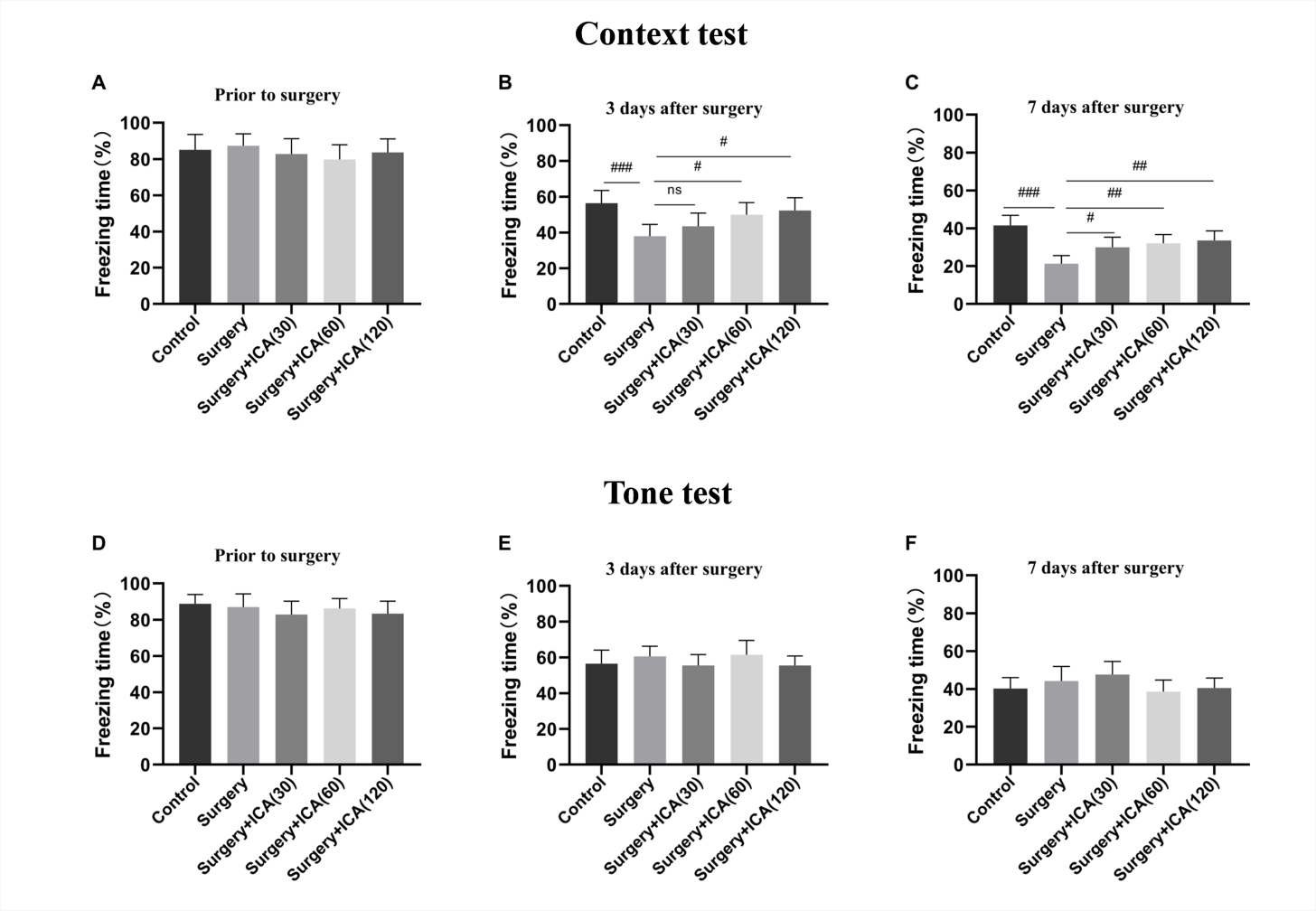


**Supplementary Figure 2**. Surgery caused Cognitive impairment and Icariin can improve cognitive impairment of POCD rats. Surgery impaired hippocampal-dependent memory, but not hippocampal-independent memory. Icariin treatment alleviated postoperative hippocampal-dependent memory impairment. (**A-C**) In the context test of FCT, the freezing time prior to surgery showed no significant differences between the groups. Surgery decreased the freezing time on postoperative days 3 and 7, as compared to the control group. Icariin treatment increased the freezing time in the context test on postoperative days 3 and 7, as compared to surgery group. (**D-F**) In the tone test of FCT, there was no statistical difference in the freezing time among five groups at all the preoperative and postoperative time points. The data are plotted as the mean ± standard deviation for each group (n = 6 per cohort). *^#^p* < 0.05; *^##^p* < 0.01; *^###^p* < 0.001.

**Conclusions**

Icariin can improve cognitive impairment induced by surgery.

**References**

Jiang, X., Chen, L.L., Lan, Z., Xiong, F., Xu, X., Yin, Y.Y., et al. (2019). Icariin Ameliorates Amyloid Pathologies by Maintaining Homeostasis of Autophagic Systems in Abeta(1-42)-Injected Rats. *Neurochem Res* 44(12)**,** 2708-2722. doi: 10.1007/s11064-019-02889-z.

Liu, Q., Sun, Y.M., Huang, H., Chen, C., Wan, J., Ma, L.H., et al. (2021). Sirtuin 3 protects against anesthesia/surgery-induced cognitive decline in aged mice by suppressing hippocampal neuroinflammation. *J Neuroinflammation* 18(1)**,** 41. doi: 10.1186/s12974-021-02089-z.

Lu, Y., Xu, X., Dong, R., Sun, L., Chen, L., Zhang, Z., et al. (2019). MicroRNA-181b-5p attenuates early postoperative cognitive dysfunction by suppressing hippocampal neuroinflammation in mice. *Cytokine* 120**,** 41-53. doi: 10.1016/j.cyto.2019.04.005.
