# Supplementary figures and images for: Icariin reduces cognitive dysfunction induced by surgical trauma in aged rats by inhibiting hippocampal neuroinflammation
Source: Front Behav Neurosci. 2023 Jun 7;17:1162009. doi: 10.3389/fnbeh.2023.1162009 (PMC10282654; doi:10.3389/fnbeh.2023.1162009)

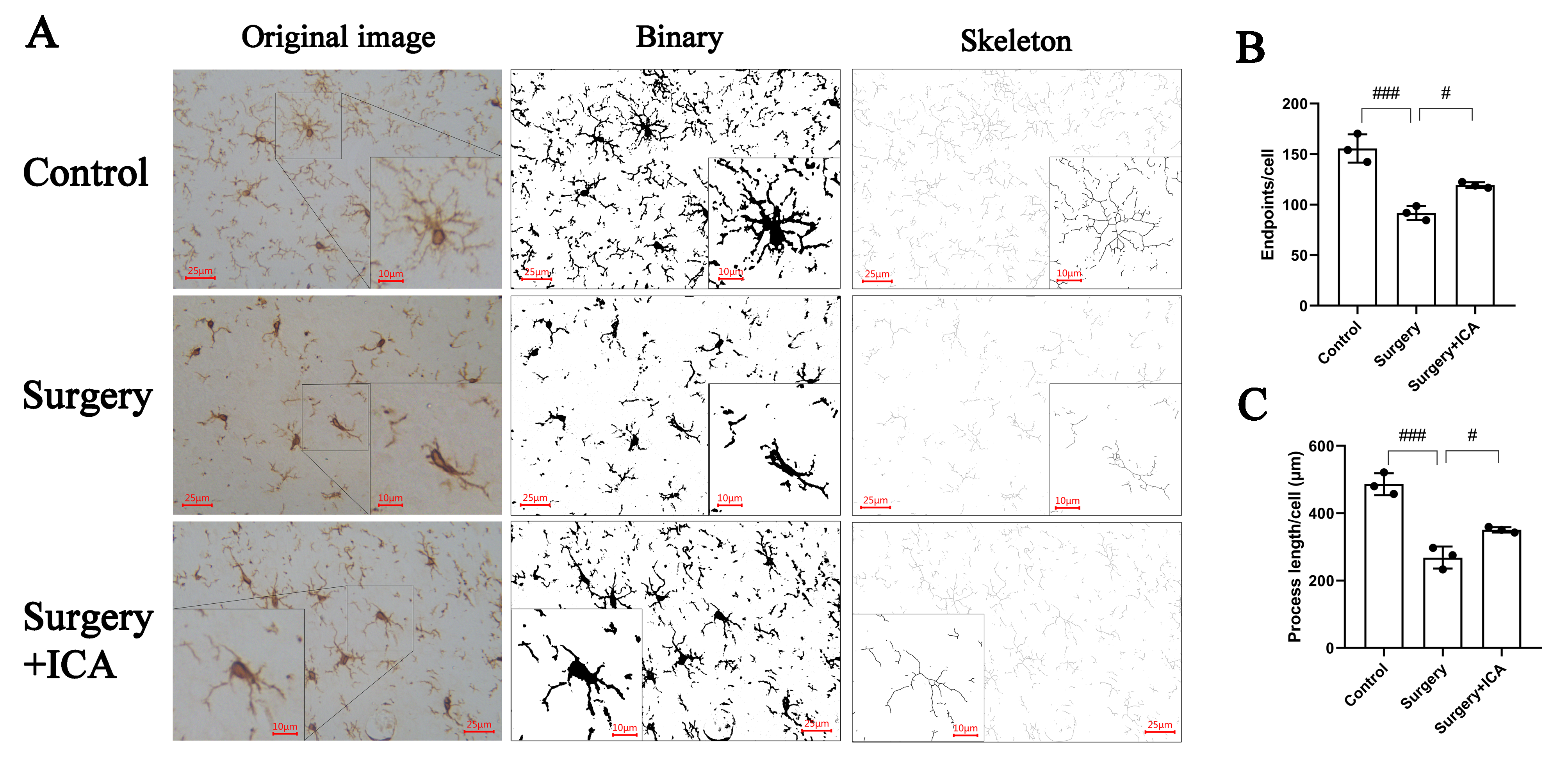

Supplement: Supplementary file 2 [file Image_1.TIF]
